# Prehospital critical care for out-of-hospital cardiac arrest: An observational study examining survival and a stakeholder-focused cost analysis

**DOI:** 10.1186/s12873-016-0109-y

**Published:** 2016-12-07

**Authors:** Johannes von Vopelius-Feldt, Jane Powell, Richard Morris, Jonathan Benger

**Affiliations:** 1 Faculty of Health and Life Sciences, University of the West of England, Glenside Campus, Blackberry Hill, Bristol, BS16 1DD UK; 2 Academic Department of Emergency Care, University Hospitals Bristol NHS Foundation Trust, Upper Maudlin Way, Bristol, BS2 8HW UK; 3 School of Social and Community Medicine, University of Bristol, Canynge Hall, 39 Whatley Road, Bristol, BS8 2PS UK

**Keywords:** Heart Arrest, Emergency Medical Services, Critical care

## Abstract

**Background:**

Survival rates from out-of-hospital cardiac arrest (OHCA) remain low, despite remarkable efforts to improve care. A number of ambulance services in the United Kingdom (UK) have developed prehospital critical care teams (CCTs) which attend critically ill patients, including OHCA. However, current scientific evidence describing CCTs attending OHCA is sparse and research to date has not demonstrated clear benefits from this model of care.

**Methods:**

This prospective, observational study will describe the effect of CCTs on survival from OHCA, when compared to advanced-life-support (ALS), the current standard of prehospital care in the UK. In addition, we will describe the association between individual critical care interventions and survival, and also the costs of CCTs for OHCA.

To examine the effect of CCTs on survival from OHCA, we will use routine Utstein variables data already collected in a number of UK ambulance trusts. We will use propensity score matching to adjust for imbalances between the CCT and ALS groups. The primary outcome will be survival to hospital discharge, with the secondary outcome of survival to hospital admission.

We will record the critical care interventions delivered during CCT attendance at OHCA. We will describe frequencies and aim to use multiple logistic regression to examine possible associations with survival.

Finally, we will undertake a stakeholder-focused cost analysis of CCTs for OHCA. This will utilise a previously published Emergency Medical Services (EMS) cost analysis toolkit and will take into account the costs incurred from use of a helicopter and the proportion of these costs currently covered by charities in the UK.

**Discussion:**

Prehospital critical care for OHCA is not universally available in many EMS. In the UK, it is variable and largely funded through public donations to charities. If this study demonstrates benefit from CCTs at an acceptable cost to the public or EMS commissioners, it will provide a rationale to increase funding and service provision. If no clinical benefit is found, the public and charities providing these services can consider concentrating their efforts on other areas of prehospital care.

**Trial registration:**

ISRCTN registry ID ISRCTN18375201.

## Background

### Prehospital treatment of out-of-hospital cardiac arrest

Sudden death due to out-of-hospital cardiac arrest (OHCA) remains a major health issue with an estimated 275,000 cardiac arrests in Europe each year [1]. Survival rates of between 5% and 38% have been reported, and have been linked to differences in prehospital treatment [1, 2]. Optimising care for patients suffering OHCA through early recognition and improved prehospital provider response times has been the focus of many emergency medical systems (EMS) over the last two decades [2, 3]. A range of different interventions has been studied [4, 5] but only early chest compressions [6, 7] and early defibrillation [2, 8] have been shown consistently to improve survival from OHCA. Despite a lack of clear evidence, many EMS have also established targeted dispatch of physicians and/or specialised critical care paramedics to OHCA; a concept referred to as prehospital critical care [9, 10]. These critical care teams (CCTs) attend cases of OHCA in addition to the standard ambulance response of paramedics trained in advanced life support (ALS). This has potential benefits, but also additional costs.

### Prehospital critical care for OHCA

The mechanisms by which outcomes might be improved through the presence of prehospital critical care practitioners include ALS interventions undertaken more efficiently, supplementation of existing protocols with enhanced experience and clinical judgment and an advanced level of post-arrest treatment [11]. In addition to these direct potential benefits of prehospital critical care, it might also allow for transport of patients with return of spontaneous circulation (ROSC) over greater distances, resulting in more patients receiving care at specialist centres, which has been shown to be beneficial [12]. There is little research addressing the concept of prehospital critical care in general, and in the context of OHCA specifically. A recently published systematic review on the impact of paramedic-delivered prehospital critical care did not identify any studies relating to OHCA [13]. A systematic review by Botker from 2009 examined the effect of physician-delivered prehospital critical care on OHCA outcomes and found a benefit, ‘based on limited evidence’ [14].

We have examined each of the five studies included in Botker’s review and have identified significant limitations in each of them. Small sample size [15] comparison of prehospital critical care with very limited basic life support [16] and study designs which did not control for significant confounding factors [16–19] make the interpretation and applicability of these findings problematic.

Olasveengen et al. acknowledged this lack of evidence regarding prehospital critical care for OHCA [20]. In 2009, the authors compared survival rates from OHCA with prehospital physician care (*n* = 232) and with paramedic ALS care (*n* = 741) in Norway. Data were collected prospectively and, after logistic regression to adjust for prognostic factors, no significant difference in outcomes was found.

In summary, there is no existing evidence to support paramedic-delivered prehospital critical care for patients with OHCA, while for physician-delivered prehospital critical care studies have shown mixed results, with the largest and most recent study failing to demonstrate any benefits [14, 20].

Research undertaken as a pilot study for this project examined the impact of the South West Ambulance Service NHS Foundation Trust’s (SWAST) CCT on survival from OHCA [21]. This retrospective database analysis compared survival to hospital discharge between 165 cases of OHCA attended by CCTs and 1686 cases attended by ALS-paramedics. While survival was significantly higher in the CCT group than the ALS-paramedic group (15.8% and 6.5%, respectively, *p* < 0.001), this was largely due to an imbalance in prognostic factors. After adjusting for these, using multiple logistic regression, there was no significant benefit from CCT attendance in this sample (OR 1.54, 95% CI 0.89–2.67, *p* = 0.13).

Currently, all EMS in the UK dispatch paramedics trained in advanced life support (ALS) to confirmed or suspected OHCA [3]. Prehospital critical care teams (CCTs) are utilised by some but not all NHS ambulance trusts, and their availability varies significantly across regions [9, 22]. This results in large variations in the care and resources available for OHCA, possibly reflecting the uncertainty regarding the clinical benefits of CCTs [9, 23].

### Economic considerations regarding prehospital treatment of OHCA

Research addressing cost-effectiveness of prehospital interventions is rare; a systematic review by Lerner in 2006 identified 32 publications, of which only two met criteria for high-quality economic evaluations [24]. A major obstacle to achieving such high-quality economic evaluations is the general paucity of high-level evidence of effectiveness, such as randomized controlled trials, in prehospital care [25]. Ten of the economic evaluations identified in the review address the factors which have been shown to improve survival from OHCA [24]. Table 1 summarises the interventions and cost per life saved found by studies included in the systematic review by Lerner et al [24]. It shows that existing interventions vary widely in their cost effectiveness, between individual interventions but also for the same intervention implemented in different circumstances. There is no publication to our knowledge that examines the provision of prehospital critical care for OHCA.

**Table 1:** Key features of publications included by Lerner et al. [24] which address the cost-effectiveness of pre-hospital interventions for OHCA

Publications	Intervention	Incremental cost: Effectiveness $/Life saved
Ornato (1988) Jackobsson (1987)	Basic life support providers	$2,800–12,900
Hallstrom (1981) Nichol (1998) Jermyn (2000) Forrer (2002) Nichol (2003)	Defibrillation (pre-hospital provider and/or lay responders)	$7,800–$190,000
Nichol (1996)	Reducing EMS response time for OHCA	$262,700–$1,134,400
Urban (1981) Valenzuela (1990)	Advanced cardiac life support providers	$91,900–$181,000

### Aims and objectives

This prospective, observational study will describe the effect of CCTs on survival from OHCA, when compared to advanced-life-support (ALS), the current standard of prehospital care in the UK. In addition, we will describe the association between individual critical care interventions and survival. A stakeholder-focused cost analysis will describe the costs of CCTs for OHCA, in relation to public donations to charity and EMS commissioning.

## Methods

All included NHS ambulance trusts dispatch prehospital critical care teams to confirmed or suspected OHCA. While the CCTs will aim to attend most cases of OHCA, they will inevitably be unavailable for a proportion of these, due to already having been dispatched to another case of critical illness or injury, or the limitations imposed by operational duty hours and constrained funding. This results in a natural experiment where one group of patients is attended by the CCT and another is treated by ALS-trained paramedics. By comparing rates of survival to hospital discharge between these two groups, we will be able to measure the impact of prehospital critical care.

### Research sites

Study recruitment will take place in up to four NHS ambulance trusts. This combination will cover both rural and urban areas of the UK. All four ambulance trusts dispatch CCTs to OHCA by helicopter or in a rapid response vehicle, depending on time of day, weather and geographical considerations. We have shown in our previous research that prehospital critical care competencies are few in number, and of these few, only a fraction apply to prehospital critical care for OHCA [10]. We therefore anticipate that the team composition, critical care competencies and relevant protocols vary only slightly between the CCTs of the different trusts. These slight differences will not influence the results to any important degree regarding the impact of prehospital critical care (as a concept) on survival. We will compare CCT protocols prior to data collection, and the individual critical care interventions delivered for each case of OHCA will be recorded.

### Inclusion/ exclusion criteria

Patients will be 18 years or older and have suffered a non-traumatic OHCA. Inclusion criteria are adult cases of OHCA where EMS providers commenced cardiopulmonary resuscitation (CPR). Excluded are cases of OHCA due to trauma, drowning, electrocution and asphyxia or OHCA where no resuscitation attempts were made. Also excluded will be cases with incomplete documentation of matching variables. We will record all excluded cases and present the inclusion/exclusion process in the Utstein format [26].

### Data collection

We will analyse consecutive cases of adult OHCA, according to the inclusion and exclusion criteria. The study is planned to commence in September 2016 and continue for 18 months or until 6000 cases have been included, whichever occurs first.

Data collection will be from two data sources.

Ambulance trusts in the UK collect OHCA data routinely for quality assurance, independently of this research. These data are based largely on the Utstein variables and are recorded in regional databases. Participating ambulance trusts will provide data from their databases to the researchers. This first data source includes the following variables required for this research project:

- ▪ Medical identifying number
- ▪ Date and time
- ▪ Location (public place, private location or nursing home)
- ▪ Postcode of event (district level)
- ▪ Age-group and gender of patient
- ▪ Do Not Attempt Resuscitation order in place
- ▪ EMS chest compressions
- ▪ Witnessed event
- ▪ Bystander CPR
- ▪ Public access defibrillator used by bystander
- ▪ Suspected cause
- ▪ First EMS resource response time
- ▪ First recorded cardiac rhythm
- ▪ ROSC on arrival at hospital
- ▪ Receiving hospital (if transported to hospital)
- ▪ Survival to hospital discharge

In addition, we will collect data from the critical care teams (CCTs). Most CCTs keep a local electronic database of all cases where the CCT was requested, including the broad category of event (for example trauma, OHCA, medical). We will integrate a few additional data requests for every case identified as OHCA, specifically for this research:

- ▪ Medical identifying number
- ▪ Stand down prior to arrival at patient
- ▪ CCT requested by ALS ambulance crew on scene
- ▪ CCT members: Critical care paramedic and/or doctor
- ▪ CCT response time
- ▪ Interventions delivered during cardiac arrest
- ▪ Interventions delivered after return of spontaneous circulation (ROSC)
- ▪ Transport decisions and modalities
- ▪ Provider decision to not provide full critical care in patient’s best interest

If no electronic CCT database exists, the required data will be collected on a simple paper form for each CCT OHCA case. The ambulance trust database and CCT data from each participating ambulance trust will be merged using the medical identifying number, creating the CCT and ALs groups.

### Data analysis

In participating ambulance trusts, dispatch of CCTs is via a dedicated procedure which assesses all incoming 999 calls for signs of critical illness or injury. The decision to dispatch the CCT is generally based on a combination of clinical decision making of the dispatcher and the CCT as well as fixed dispatch criteria. In some cases EMS providers will request support from a CCT after arriving on scene. Our pilot study showed that the CCT is more likely to be dispatched to OHCAs with good prognostic factors, such as an OHCA with bystander CPR in a younger patient. The overall patient group attended by a CCT therefore has more favourable prognostic factors compared to the group attended by ALS-trained paramedics. To correct for this imbalance, we will match cases of the CCT group with controls of the ALS-trained paramedic group, using propensity score matching. This method of controlling for prognostic variables in observational data has been used successfully in OHCA research recently [27, 28]. We have shown that CCTs attend approximately 10% of all OHCA cases, resulting in a large pool of controls, allowing us to match multiple controls with each case (see step 2 below).

Propensity score matching will involve the following steps [29]:

1. We will run a logistic regression with CCT attendance as the dependent variable. Prognostic factors for both survival to hospital discharge and CCT dispatch will be covariates. They will include age and gender of the patient, the cause and location of OHCA, whether it was witnessed, bystander CPR, ambulance response time, first recorded rhythm and distance from CCT base. This will allow us to calculate a propensity score for each patient - the probability of the patient receiving CCT treatment.
2. We will then match each CCT case with ALS-trained paramedic cases of OHCA, with similar propensity scores. This will be done using nearest neighbour matching within a specified calliper distance - the ‘greedy algorithm’ assigns controls with similar propensity scores to each case. To qualify as 'matching' the propensity score of potential controls needs to be within 0.2 of the pooled standard deviation of the logit of the propensity score [30]. This reduces the risk of bias from matching controls to cases whose propensity scores differ too much. To optimise available data, we will match each CCT case with up to five ALS-paramedic controls whose propensity scores are within the defined caliper, accepting varying numbers of controls per case. Each control will only be available for matching once and then will be removed from the control pool [31].
3. We will compare the matched CCT and ALS-paramedic groups to verify that prognostic factors are balanced between the groups.
4. We will analyse for statistically significant differences in survival to hospital discharge between the groups (see also ‘3.2.5 data analysis’).

The matching process will result in two groups of OHCA patients: an intervention group that has been attended by a CCT in addition to the usual ambulance response, and a control group that has been attended by ALS-trained paramedics alone. We will compare the primary outcome of survival to hospital discharge between OHCA attended by the CCT and OHCA attended by ALS-trained paramedics. We will conduct multivariable logistic regression analyses for the full cohort and conditional logistic regression for the propensity score matched groups. We will also undertake subgroup analysis for witnessed OHCA with shockable rhythm, with the hypothesis that any potential benefit of CCT attendance will be more pronounced. There is a possibility that any observed benefit from CCT care is due partially to CCTs conveying more patients to a cardiac centre, bypassing nearer hospitals. We will therefore also compare the secondary outcome of survival to hospital admission.

In addition, we will describe the frequency of prehospital critical care interventions delivered by the CCTs for OHCA. We will then undertake logistic regression analysis including known predictors of survival, with the individual critical care interventions as independent variables and survival to hospital discharge as a dependent variable. We estimate that there will be enough survivors of OHCA to allow multiple logistic regression with up to ten variables (estimated 100 survivors, with 10 outcomes per variable). However, should this not be possible, we will describe frequency of critical care interventions occurring during OHCA treatment, which will be useful information when interpreting the overall results of this project.

### Sample size

Data for the South Western Ambulance Service from our pilot study shows that resuscitation for OHCA was commenced by prehospital providers in approximately 100 cases during the month. Rates of survival average 7.5%. We estimate that after application of inclusion and exclusion criteria, we will be able to include 6000 patients, of which approximately 600 will be CCT cases. This will allow us to detect an absolute improvement in survival rates of approximately 4.5% with a power of 0.8 and alpha 0.05, assuming one-to-two matching and a survival rate of 7.5% in the control group.

### Stakeholder-focused cost analysis

A cost analysis of prehospital critical care for OHCA will be undertaken for the South Western Ambulance Service NHS Foundation Trust (SWAST). We will follow a previously published and validated structure for cost analyses of EMS [32]. The toolkit treats resource use and prices separately from a societal perspective. Cost analysis is undertaken using the following steps:

1. Define the community for which costs are being calculated
2. Determine all of the agencies that are part of the EMS system
3. Estimate the percentage of time that each agency is involved in EMS
4. Calculate human resource costs
5. Calculate physical plant costs
6. Calculate vehicle costs
7. Calculate equipment costs
8. Calculate other administrative costs
9. Calculate EMS-related training costs
10. Calculate bystander training costs
11. Calculate any costs associated with revenue generation

The necessary data for the cost calculations are available from routine billing documents of SWAST and the Great Western Air Ambulance Charity. Other important information, such as average number of paramedics and vehicles dispatched to OHCA and time spent with each case will be extracted from the SWAST OHCA database and routinely collected data. We will calculate the costs of providing ALS-trained paramedic prehospital care for OHCA and the incremental costs of providing physician-led prehospital critical care. Combining this incremental cost with the impact of CCTs on survival to hospital discharge after OHCA will allow decision-makers in EMS and the public donating money to air ambulance charities to assess the cost-effectiveness of prehospital critical care for OHCA. In addition to the total incremental costs of CCTs, we will also present the costs currently covered by charities and those currently covered by NHS organisations. We will also differentiate between costs caused by utilisation of a helicopter and use sensitivity analysis to estimate the impact of transport modality on overall costs. Our approach to costing is pragmatic. We will not describe the costs of in-hospital or post-discharge treatments, as they are unlikely to influence stakeholders in EMS commissioning.

## Discussion

The overall aim of this research project is to guide the commissioning of prehospital care for OHCA. As such, the research focuses on the clinical effect of prehospital critical care teams, as well as its costs, as far as EMS spending is concerned. In the UK, a large part of prehospital critical care is currently supported by the public, through donations to charities. Understanding how these different groups support and interpret relevant research is therefore important to maximise the impact of future research.

The ideal outcome to measure the success of prehospital interventions for OHCA is unclear. Early outcomes such as ROSC, while generally clearly attributable to EMS interventions, are not patient-focused and can overestimate the actual benefits of interventions that might only prolong the interval between OHCA and the patient’s subsequent unavoidable death [4]. The ideal patient-focused outcome after OHCA would be long-term survival with good neurological function [33]. The disadvantages of using this outcome are the resources required to record it as well as loss of patients to follow up. In addition, patients with long term survival will have spent minutes in the EMS, followed by days in hospital, which raises the issue of differences in hospital treatment confounding results [33, 34]. As a pragmatic approach, we chose survival to hospital discharge as the primary outcome, and survival to hospital admission as a secondary outcome. Survival to hospital discharge in the UK is associated with a high likelihood of survival with normal or mildly impaired neurological status (89% in a study of 1476 patients admitted to hospital after OHCA) [35]. We therefore consider it a satisfactory surrogate outcome for this study.

The research is designed as a multicentre study, which will help generalisability. The different prehospital critical care services participating in this research will all have slight variation in their deployment (helicopter and/or rapid response vehicle) and the interventions that are used when treating OHCA. After review of local and regional practice, we are assured that these differences are not large enough to limit the generalisability of our findings. In fact, the variation in interventions between cases of OHCA treated within the same CCT observed in our pilot analysis is much more significant, ranging from zero to eight critical care interventions per case. To account for these variations, we will record all critical care interventions delivered for each case.

This research aims to inform relevant stakeholders in prehospital care of the effects and costs of prehospital critical care following OHCA. Together with a detailed description of critical care interventions undertaken during the care of patients who suffer OHCA, this information can guide future practice and funding of prehospital care for OHCA. The results will be made publicly available on an open access website, and we will publish the findings in appropriate journals and present them at national and international conferences relevant to the subject field.

## Abbreviations

ALS: Advanced life support
CCT: Critical care team
CPR: Cardiopulmonary resuscitation
EMS: Emergency Medical Service
OHCA: Out-of-hospital cardiac arrest
ROSC: Return of spontaneous circulation
SWAST: South Western Ambulance Service NHS Foundation Trust
UK: United Kingdom
